# Temporal trend of breast cancer burden among younger and older Brazilian women, 1990–2019

**DOI:** 10.1590/1980-549720250006

**Published:** 2025-03-03

**Authors:** Thayane Duarte Silva Santos, Camila de Araújo Gonçalves, Camila Petronilia da Cunha, Jéssica Patrocínio Milhomem, Kriscylla Magalhães da Silva, Bruno Teixeira da Costa, Rafaela Galdeano Piantolo, Raphael Joaquim Couto Fernandes, Yuri Marques da Silva, Raphael Mendonça Guimarães

**Affiliations:** I Universidade Estácio de Sá, School of Medicine – Rio de Janeiro (RJ), Brazil.; II Oswaldo Cruz Foundation, National School of Public Health – Rio de Janeiro (RJ), Brazil.

**Keywords:** Epidemiology, Breast neoplasms, Female, Cost of illness, Time series studies, Epidemiologia, Neoplasias da mama, Feminino, Efeitos psicossociais da doença, Estudos de séries temporais

## Abstract

**Objective:**

To analyze the temporal trend of the burden of breast cancer in Brazilian women under 40 years of age compared to the age group over 40 years of age, between 1996 and 2019.

**Methods:**

An ecological time trend study was conducted in Brazil between 1996 and 2019 using data from the Global Burden of Disease (GBD) study. The segmented regression method (Joinpoint Regression) was applied to analyze rates among women under and over 40 years of age. To capture differences in the level and trend of mortality and DALYs, the rate ratio was calculated for the two groups on a year-by-year basis.

**Results:**

Regarding DALY, an average annual decline of 0.7% (95%CI −0.8 to −0.5, p<0.01) was observed among women over 40 years old, while an annual increase of 1.0% (95%CI 0.9 to 1.1, p<0.001) was noted for women up to 40 years old. For mortality, the decline among older women was 0.3% per year (95%CI −0.4 to −0.2, p<0.001), and the increase among young women was 0.8% per year (95%CI 0.7 to 1.0, p<0.001). The average rate ratio for DAILY was 5.2, while for mortality, the average rate ratio was 8.1.

**Conclusion:**

the analysis reinforces the idea that the magnitude and trend of breast cancer mortality among young women is a health issue requiring attention from health decision-makers. This diagnosis underscores the importance of initiating discussions on the need to review population screening criteria, incorporating clinical prediction rules.

## RESUMO

**Objetivo:**

Analisar a tendência temporal da carga de câncer de mama em mulheres brasileiras com menos de 40 anos em comparação à faixa etária acima de 40 anos, entre 1996 e 2019.

**Métodos:**

Realizou-se um estudo ecológico de tendência temporal no Brasil entre 1996 e 2019, a partir de dados do estudo *Global Burden of Disease* (GBD). O método de regressão segmentada (*Joinpoint Regression*) foi aplicado para analisar as taxas entre mulheres abaixo e acima de 40 anos. Para capturar as diferenças entre o nível e a tendência da mortalidade e dos DALYs, calculou-se a razão de taxas ano a ano para os dois grupos.

**Resultados:**

Em relação ao DALY, observou-se um declínio médio anual de 0,7% (IC95% −0,8 a −0,5, p<0,01) para mulheres acima de 40 anos e um aumento de 1,0% ao ano (IC95% 0,9 a 1,1, p<0,001) para mulheres até 40 anos. Para a mortalidade, o declínio entre as mulheres mais velhas é de 0,3% ao ano (IC95% −0,4 a −0,2, p<0,001), e o aumento entre as mulheres jovens é de 0,8% ao ano (IC95% 0,7 a 1,0, p<0,001). A razão média de taxas para DALYs foi de 5,2, enquanto para a mortalidade foi de 8,1.

**Conclusão:**

A análise reforça a ideia de que a magnitude e a tendência da mortalidade por câncer de mama entre mulheres jovens constituem uma questão de saúde que requer atenção dos tomadores de decisão. Este diagnóstico destaca a importância de iniciar discussões sobre a necessidade de revisar os critérios de rastreamento populacional, incorporando regras de predição clínica.

## INTRODUCTION

Breast cancer is the most common cancer among women worldwide, with 2.29 million new cases diagnosed in 2022. The disease is responsible for 667,000 deaths annually, making it the fifth leading cause of cancer deaths in women^1^. In Brazil, breast cancer is the most common cancer among women. The National Cancer Institute in Brazil estimates that 73,610 new breast cancer cases will be diagnosed in 2023, with an incidence rate of 41.89 cases per 100,000 women^2^.

The mortality rate from breast cancer in Brazil increases progressively with age. However, the mortality rate among women under 40 is not negligible. In 2021, the mortality rate from breast cancer in women under 40 years of age in Brazil was 2.47 deaths/100,000 women^3^. This accounts for approximately 7% of total deaths from this type of cancer.

It is essential to recognize that, following diagnosis, young women face a greater risk of psychosocial issues. This is due not only to a less favorable clinical condition but also to the stage of life at the time of diagnosis and specific concerns for young breast cancer patients, such as fertility preservation, family planning, sexual function, perception of beauty, and body image^4^. For this reason, analyzing data for young women can contribute to a more comprehensive understanding of the breast cancer mortality scenario in the country, helping to identify priority target populations for the revision of Breast Cancer Screening guidelines. Thus, this study aimed to analyze the temporal trend of the burden of breast cancer in Brazilian women under 40 years of age, compared to women over 40 years of age, between 1996 and 2019.

## METHODS

An ecological time trend study was conducted to assess Brazil's breast cancer burden between 1996 and 2019, using data from Brazil to perform the analysis. We used the dataset from the Global Burden of Diseases (GBD) study. The GBD study estimates the burden of disease for a range of causes, risk factors, and covariates, such as gender and age, across several countries, including subnational analyses. A detailed description of the national data and methodology used to assemble the prediction is further provided by the Global Burden of Disease 2019 Cancer Collaboration^5^.

Data on both mortality and burden of breast cancer among women in Brazil were extracted. Mortality rates refer to deaths from breast cancer relative to the population at risk, while the Disability-Adjusted Life Year (DALY) metric quantifies the global burden of disease by combining years of life lost due to premature death (YLL) and years lived with disability (YLD). One DALY is equivalent to one year of healthy life lost due to disease or death.

The segmented regression method (Joinpoint Regression) was applied to identify significant changes in the trend over the period. This model assumes a linear trend between the inflection points (joinpoints). The joinpoint model for the observations, (x_1_, y_1_), …, (x_n_, y_n_), where x_1_ ≤ … ≤ x_n_ is defined as:


$$\text{E}[\text{y|x}]={\text{β}}_{0}+{\text{β}}_{1}\text{x}+{\text{δ}}_{1}{\left(\text{x}-{\text{τ}}_{1}\right)}^{+}+\ldots +{\text{δ}}_{\text{k}}{\left(\text{x}-{\text{τ}}_{\text{k}}\right)}^{+}$$

where τ_k_'s are unknown joinpoints and a^+^ = a for a > 0 and 0 otherwise (6).

To ensure the assumption of homoscedasticity, Poisson distribution parameters with robust variance were used.

To estimate annual percentage change (APC), the following model was applied:


$$\log\left({\text{Y}}_{\text{x}}\right)={\text{β}}_{0}+{\text{β}}_{1}\text{x}$$

where log (Y_x_) is the natural logarithm of the rate in year x.

Then, the APC from year x to year x + 1 is:


$$\text{APC}=\frac{{\text{e}}^{{\text{β}}_{0}+{\text{β}}_{1}(\text{x}+1)}-{\text{e}}^{{\text{β}}_{0}+{\text{β}}_{1}\text{x}}}{{\text{e}}^{{\text{β}}_{0}+{\text{β}}_{1}\text{x}}}\times 100=\left({\text{e}}^{{\text{β}}_{1}}-1\right)\times 100$$

A 95% confidence interval was estimated considering:


$$\begin{array}{l} {\text{APC}}_{\text{L}}=\left({\text{e}}^{\log(\text{APC}+1)-1,96\sqrt{{\text{w}}_{\text{x}}^{2}{\text{σ}}_{\text{x}}^{2}})}-1\right)\times 100;\\ {\text{APC}}_{\text{U}}=\left({\text{e}}^{\log(\text{APC}+1)+1,96\sqrt{{\text{w}}_{x}^{2}{\text{σ}}_{x}^{2}})}-1\right)\times 100 \end{array}$$

where σ^2^_x_ is the estimate of the variance of ßx obtained from the adjustment of the joinpoint model. Once the number k of joinpoints is defined, different models with k joinpoints are compared by estimating their Bayesian Information Criterion (BIC)^6^.

Finally, to capture differences between the level and trend of mortality and DALYs for breast cancer in young women (up to 40), comparisons were made with the level and trend of women over 40. The differences were estimated based on the rate ratio for the two groups, year by year. In the same way, as the groups were analyzed separately, the trend in the rate ratios for the groups was explored. A 5% alpha level was considered significant, and the 95% confidence interval (95%CI) was estimated for all calculations.

This research used secondary public data, with no risk of identification. In accordance with Brazilian legislation on research involving human subjects (Resolution 466/2012 and 510/2016), the project is exempt from review by a research ethics committee.

## RESULTS

**Figure 1:**
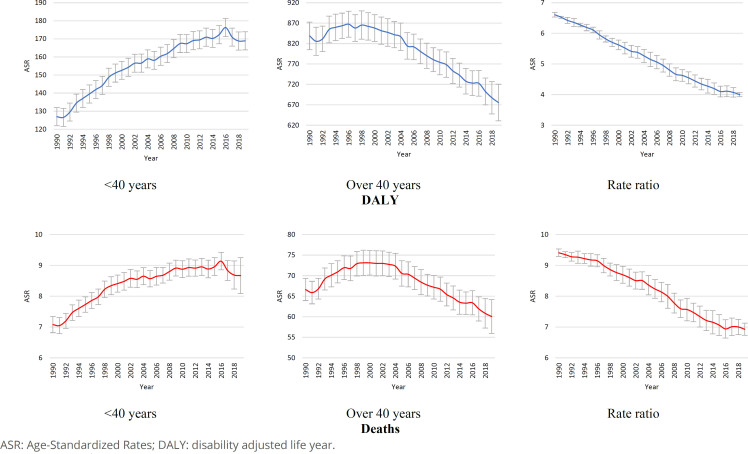
Time series of breast cancer age-standardized disability adjusted life year and mortality rate among women below 40 years and over 40 years. Brazil, 1990––2019.

Source: GBD study, 2023.

The decomposition of disease burden indicators reveals a significant difference in the behavior of breast cancer between women under 40 years of age and those over 40 years of age (Figure 1). Whether for the mortality rate or the DALY rate, there is a downward trend for women over 40 and an upward trend for the group up to 40 years old. It is important to note that the magnitude between the groups is highly unequal, as evidenced by the rate ratio. For DALY, the average rate ratio is 5.2, while for mortality, the average rate ratio is 8.1. This highlights the greater magnitude of the impact of breast cancer on older women.

**Figure 2:**
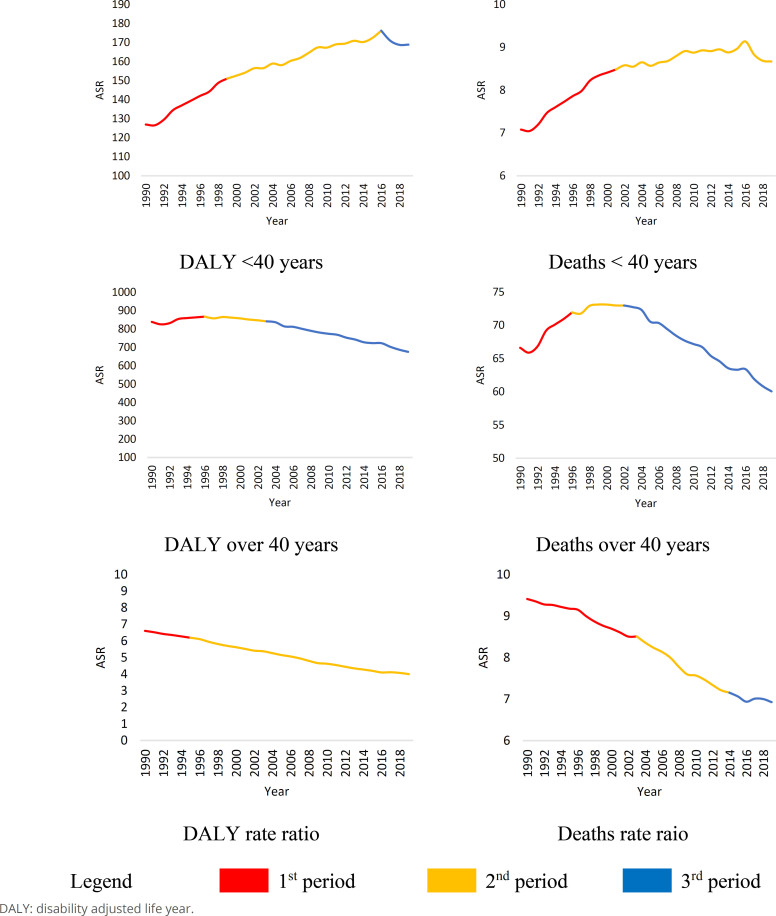
Time segments for breast cancer age-standardized disability adjusted life year and mortality rate among women below 40 years and over 40 years. Brazil, 1990–2019.

Source: GBD Study, 2023

Despite the substantial difference in magnitude between the groups, the temporal trend demonstrates the relevance of studying breast cancer among young women (Figure 2 and Table 1). Regarding DALY, there is an average annual decline of 0.7% (95%CI −0.8 to −0.5, p<0.01) for women over 40 years old and an annual increase of 1.0% (95%CI 0.9 to 1.1, p<0.001) for women up to 40 years old. For mortality, the decline among older women is 0.3% per year (95%CI −0.4 to −0.2, p<0.001), while the increase among young women is 0.8% per year (95%CI 0.7 to 1.0, p<0.001).

**Table 1:** Joinpoint periods for breast cancer age-standardized disability adjusted life year and mortality rate among women below 40 years and over 40 years. Brazil, 1990–2019.

Indicator	Group	Period	APC	95%CI	p-value
DALY	<40 years	1990–1999	2.1	(1.9 to 2.4)	<0.001
		1999–2016	0.8	(0.7 to 0.9)	<0.001
		2016–2019	-1.5	(-2.8 to −0.6)	0.002
		AAPC	1.0	(0.9 to 1.1)	<0.001
	Over 40 years	1990–1996	0.8	(0.4 to 1.2)	<0.001
		1996–2003	-0.4	(-0.8 to 0.0)	0.051
		2003–2019	-1.3	(-1.4 to −1.2)	<0.001
		AAPC	-0.7	(-0.8 to −0.5)	<0.001
	Rate ratio	1990–1995	-1.3	(-1.9 to −0.7)	<0.001
		1995–2019	-1.9	(-2.0 to −1.7)	<0.001
		AAPC	-1.8	(-1.9 to −1.7)	<0.001
Deaths	<40 years	1990-2001	1.9	(1.6 to 2.1)	<0.001
		2001-2019	0.2	(0.1 to 0.3)	0.002
		AAPC	0.8	(0.7 to 1.0)	<0.001
	Over 40 years	1990-1996	1.5	(1.2 to 2.1)	<0.001
		1996-2002	0.3	(-0.2 to 0.7)	0.147
		2002-2019	-1.2	(-1.3 to −1.1)	<0.001
		AAPC	-0.3	(-0.4 to −0.2)	<0.001
	Rate ratio	1990-2003	-0.8	(-1.0 to −0.7)	<0.001
		2003-2014	-1.6	(-1.8 to −1.5)	<0.001
		2014-2019	-0.4	(-0.9 to −0.1)	0.045
		AAPC	-1.1	(-1.2 to −1.0)	<0.001

APC: annual percentage change; DALY: disability adjusted life year; AAPC: average annual percentage change.

Source: GBD Study, 2023

Regarding DALY, it is noteworthy that there has been a recent decline for the group of women aged up to 40 years. This suggests a reduction in disease burden that is still developing for this group, though significant. The rate ratio for the two indicators confirms a concomitant decrease in the group aged up to 40 years and an increase in the group over 40. The comparison between DALY and mortality suggests that the trend of change is faster for DALY than for mortality. This information indicates that mortality is more consistent over time and has a prolonged change compared to the morbidity indicator. The rate ratio confirms this evidence: DALY has decreased throughout the period, which accelerated in the last 25 years. Mortality has shown only a slight slowdown in the previous five years. Although mortality continues to decline, this slowdown suggests significant changes in at least one of the two groups.

## DISCUSSION

The main results indicate that breast cancer presents different behaviors among women under 40 and over 40 years of age. For women over 40, there is a downward trend in mortality rates and DALY, with an average annual reduction of 0.7% for DALY and 0.3% for mortality. In contrast, for women under 40 years of age, mortality and DALY rates are increasing, with annual growth of 1.0% for DALY and 0.8% for mortality. The comparison between mortality and DALY indicates that changes in the DALY trend are occurring more rapidly than in mortality, with an acceleration in the reduction of DALY over the last 25 years and a slight slowdown in the decline of mortality over the past five years. These results underscore the relevance of discussing screening and early detection for breast cancer, highlighting the importance of studying breast cancer in young women due to the increase in rates within this group.

Breast cancer is one of the diseases that most affects women's health worldwide. Although the rates are lower than in older women, breast cancer mortality among young women is increasing at a faster pace. Recent studies on breast cancer in young women in Brazil^7-10^ converge on the same evidence. Notably, the study by Bonadio et al.^9^, which evaluated trends in the proportion of new breast cancer cases and deaths in patients under 40 years of age over the past decade based on data from a High Complexity Oncology Center in Brazil, showed that the contribution of young women to the incidence of breast cancer increased from 9.9% (95%CI 9.2–10.7%) between 2009 and 2014 to 12.9% (95%CI 12.1–13.8%) from 2015 to 2020. The same study also indicated an increase in mortality: 7.9% (95%CI 6.2–9.8%) in 2009 to 21.8% (95%CI 19.1–24.8%) in 2020. In a more comprehensive analysis using population-based data, Cancela et al.^10^ calculated crude breast cancer incidence rates (per 100,000 women) for women under 40 and analyzed incidence trends using segmented regression analysis. The authors observed a significant increase in incidence.

It is essential to consider that limited access to health services, such as mammography, and the low quality of health care are decisive factors for women's survival. In Brazil, the 5-year survival rate for breast cancer is 81.8%, similar to the global rate. This result reflects universal access to health care in the country, which guarantees all women the right both to free mammography and treatment^11^. In fact, the main factors that influence breast cancer survival are the stage of the disease at the time of diagnosis and the type of treatment performed.

Population screening programs for breast cancer aim to identify the disease early and provide effective treatment. The World Health Organization (WHO) recommends organized, population-based mammography screening every two years for women at an average risk of developing breast cancer, aged between 50 and 69. For women between 40 and 49 years old at high risk, and individual assessment for women over 70^12^.

Early detection plays a crucial role in survival. However, women under 40 are not included in the current guideline. Thus, these tumors are often discovered at more advanced stages and tend to be more aggressive, making treatment more challenging and reducing survival chances^13^. Breast cancer in women under 40 is often a distinct molecular biological entity. Many of these tumors are triple-negative and highly aggressive. Furthermore, these tumors are often diagnosed at advanced stages, partly due to the impossibility of tracking tumors with high duplication rates, and partly due to barriers to screening in compliance with national guidelines^14^, including young women at high risk^15^.

It is worth mentioning that mammographic screening of young women is widespread in Brazil, especially in the private health services network. However, universal recommendation of this intervention is not advisable due to a lack of evidence from clinical trials supporting its benefits for younger age ranges. Additionally, mammography in younger women has lower sensitivity and may lead to harms such as overdiagnosis, overtreatment, increased false-positive results, and risks associated with cumulative ionizing radiation from multiple screenings^16^. Both scenarios are detrimental to ensuring adequate management of young patients.

Regarding treatment, the 2000s marked a milestone in the approach to breast cancer, with the widespread adoption of taxane-based therapies and, more recently, trastuzumab, contributing to reduced mortality rates, as illustrated in the graphics. These improvements have limited impact on very aggressive tumors, which often have poor stage-related prognoses and greater resistance to traditional therapies^17,18^. State-of-the-art treatments for breast cancer in young women include targeted therapies, immunotherapy, and advances in minimally invasive surgery. Furthermore, personalized treatment based on genetic profiles allows more effective approaches, while minimizing side effects^19^.

In Brazil, health policies for breast cancer have advanced in recent decades, focusing on prevention, early diagnosis, and treatment. The National Cancer Control Policy (*Política Nacional para a Prevenção e Controle do Câncer* – PNCCM), recently launched by the Ministry of Health, has strengthened efforts to expand access to mammography for women over 50 and promoted educational campaigns on self-examination^20^. Creating specialized centers and including medications in the Unified Health System (*Sistema Único de Saúde* – SUS) have also contributed to treatment. However, challenges remain, such as regional inequality in access to exams and treatments, especially in rural and remote areas.

SUS has gradually expanded the availability of surgical treatments and incorporated new technologies in breast cancer care^21^. Mastectomy, conservative surgery, as well as breast reconstruction are available to patients in authorized health units. Immediate reconstruction after mastectomy has become increasingly common, supported by laws such as Law 12.802/2013, which guarantees the right to the procedure^22^. Furthermore, the SUS has incorporated targeted therapies and innovative treatments, including biological drugs and genetic tests, to enable personalize treatment. However, the unequal distribution of advanced technologies and prolonged waiting times for complex procedures remain critical challenges.

Although screening guidelines and programs may vary from country to country due to differences in healthcare structures, available resources, and local epidemiological research, there is general consensus on the importance of a comprehensive approach to early breast cancer detection. This means that universal coverage for a particular age group may be less efficient compared to restricted coverage after women's risk classification, regardless of their age group. In this case, individual risk would take precedence over the strictly epidemiological nexus, which considers age an essential factor in defining priorities. The data presented reinforce that age should not be considered an isolated factor when adopting screening measures. Recent trends highlight the need to create opportunities to improve the risk-benefit ratio of screening through optimizing the risk-stratified screening strategy using existing and developing risk prediction models that include women under 40 years, including the use of artificial intelligence to assess risk.

An effective approach to designing screening strategies for younger female populations is the use of clinical prediction rules. Several breast cancer risk prediction models are available to assess an individual's risk of developing malignancy or having a mutation associated with the hereditary risk of developing cancer^23^. In resource-limited settings, the adoption of clinical prediction models can facilitate access for women with higher risk potential, ensuring equity in diagnostic opportunities^24^. Similarly, these models could accurately identify groups of young women in more immediate need of rigorous monitoring or preventive measures, benefiting women at varying risk levels without necessitating the universal provision of mammography for younger women^25^.

The degree of implementation of the health policies in Brazil, including adherence by states and municipalities to screening guidelines and treatment protocols, may explain the observed DALY trend in the country. However, it is important to note that this study represents an initial approach to the topic and did not aim to stratify the analysis by state. It is hoped that this preliminary diagnosis will inspire future analyses.

In fact, there is a gap in Brazil regarding the availability of validated models for the early detection of breast cancer in young women. Although there are criteria that classify women according to risk (such as the occurrence of breast cancer in women from previous generations, presence of BRCA mutations, among others), there is no validated mathematical model for the country, with safe cutoff points for pre-test probability that indicate mammography for women outside the 50 to 69 age cutoffs. Considering the development of such a strategy for the future. In conclusion, our analysis reinforces the idea that the magnitude and trend of breast cancer mortality among young women is a health issue that deserves attention from health decision-makers.
